# The effort of maintaining the defensive wall: is there a link between defense mechanisms and vitality?

**DOI:** 10.1186/s12888-026-08146-2

**Published:** 2026-05-09

**Authors:** Anja Vandersmissen, Patrick Fissler Plischke, Marco Filippi, Maya Wenzel, Maren Bernhardt, Rainer Krähenmann

**Affiliations:** 1 Psychiatric Services Thurgau (Academic Teaching Hospital of the University of Konstanz), Spital Thurgau AG, Seeblickstrasse 3, Münsterlingen, 8596 Switzerland; 2 Department of Psychology, University of Zurich, Zurich, Switzerland; 3 Neuropsychology, University of Konstanz, Konstanz, Germany; 4 Department of Psychiatry, Psychotherapy and Psychosomatics, University of Zürich, Zürich, Switzerland

**Keywords:** Defense mechanisms, Vitality, DMRS-SR-30, Psychological distress, Coping

## Abstract

**Background:**

Vitality – the sensation of energy availability and lack of fatigue – is a transdiagnostic factor playing a role in various psychiatric disorders. Defense mechanisms are strategies individuals use to deal with internal or external stress and can be categorized by their degree of adaptiveness, summarized as defensive maturity. Immature defense mechanisms involve awareness inhibition, which is assumed to be an energy-demanding process contributing to reduced vitality. In this study, we examine whether defense mechanisms are associated with vitality.

**Methods:**

A transdiagnostic sample of 200 participants (100 with and 100 without psychiatric disorders) was analyzed in a cross-sectional design. Defensive maturity and mature and immature defense mechanisms were assessed using the Defense Mechanisms Rating Scale-Self-Report-30. Vitality was measured using the vigor and fatigue subscales of the Profile of Mood States.

**Results:**

In line with our hypotheses, defensive maturity and immature defense mechanisms were associated with vitality (*r* = .31, *p* < .001 and *r* = − .27, *p* < .001, respectively), even after adjusting for sleep quality (*β* = 0.20, *p* = .004 and *β* = − 0.16, *p* = .023, respectively). Additional analyses that accounted for both mature and immature mechanisms revealed that only immature defense mechanisms (*β* = − 0.32, *p* < .001), but not mature (*β* = − 0.07, *p* = .356), predicted vitality.

**Conclusions:**

Our results show that primarily immature defense mechanisms are related to impaired vitality. This is in line with the assumption that inhibiting thoughts or emotions is an energy-demanding process. Alternatively, a lack of energy availability may impair defense maturity. Targeting immature defense mechanisms in psychotherapeutic interventions may improve vitality as a transdiagnostic factor in a variety of psychiatric disorders in which fatigue is an accompanying symptom.

**Trial registration:**

The study was registered on 2022-07-30 on https://clinicaltrials.gov/ (identifier: NCT05544877).

**Supplementary Information:**

The online version contains supplementary material available at 10.1186/s12888-026-08146-2.

## Introduction

Defense mechanisms (DM) are automatic psychological strategies to protect individuals from the awareness of emotional conflicts, excessive anxiety, or loss of self-esteem [1–3]. DM have their roots in psychoanalysis, but they are becoming known in everyday life, too. After failing an exam, a student might explain, “The test was full of things we barely covered in class, and the teacher made it unnecessarily difficult. Many other students probably struggled, too – it wasn’t fair.” By emphasizing external factors and presumed group consensus, the student creates a reasonable-sounding explanation that protects their self-esteem and helps avoid confronting their own lack of preparation, reflecting the DM rationalization. The different DM can be sorted into three categories depending on their adaptiveness: mature, neurotic, and immature [4]. Previous research has shown that psychopathology and lower levels of psychological functioning are associated with a greater use of immature DM [5, 6], whereas mature DM and greater defensive maturity are correlated with better physical and psychological health [7–9]. Impaired vitality, i.e., fatigue or reduced feelings of vigor were found to be transdiagnostic symptoms across many psychiatric disorders [10]. Moreover, impaired vitality is associated with cognitive impairments, reduced functioning and quality of life, and has a detrimental impact on mental and physical health [11, 12]. Since disadvantageous forms of DM and impaired vitality are prevalent symptoms of several neurological and psychiatric illnesses [13], it is important to clarify how these constructs relate to one another. This article therefore aims to examine the associations of DM and vitality in a transdiagnostic sample.

Defense mechanisms mainly occur unconsciously in reaction to emotional conflicts, or internal or external stressors with the goal of protecting the individual from excessive anxiety or loss of self-esteem, for example, when something external threatens the self or after the emergence of painful or unacceptable feelings or thoughts [1–3]. All DM somehow distort reality that would otherwise be too painful to acknowledge [2]. Although there are some similarities between mature DM and coping, the differences are that DM are defined as automatic processes in reaction to emotional conflicts, or internal or external stressors [1, 3], whereas coping is defined as an active and conscious process [14, 15]. Moreover, mature DM share many characteristics with emotion regulation, which also consists of conscious processes (explicit emotion regulation) and unintentional automatic processes (implicit emotion regulation; 16].

Since the first conceptualization of defense mechanisms by Sigmund Freud, many others have contributed to their further development [17]. The gold standard classification of defense mechanisms, the Defense Mechanisms Rating Scales by Perry & Henry (DMRS), consists of 30 DM hierarchically structured on seven defense levels and three defense categories [4] that differ in the extent of their so-called adaptiveness or maturity. According to this conceptualization, individuals who rely to a greater extent on immature defenses show sparse awareness of both cognitive and emotional aspects of internal conflicts or stress since DM of this category inhibit awareness. Defenses in the neurotic defense category are characterized by focusing only on the cognitive or emotional aspects of internal conflicts or stress, because an integrated perception of the situation is too threatening. The third category of defenses, mature DM, help a person experiencing an internal conflict or stress to integrate thoughts, emotions, or desires into a mostly aware experience perceived without distortion [18].

Studies have shown that immature DM are associated with higher psychopathology and a lower level of psychological functioning [3, 5]. Moreover, longitudinal data has shown that defensive maturity predicts better physical health [7] and two studies examining patients with diabetes and breast cancer found correlations between quality of life and the use of DM [8, 19]. Healthy individuals mostly use mature or neurotic DM as a reaction to stress, but traumatic events might cause an individual to use more immature defenses [13].

We define vitality as a one-dimensional construct with fatigue and vigor (also called energy [20], or energy sensations [21]) at opposite poles. Vitality might also represent the phenomenological part of the biological energy system [21, 22]. Impaired vitality, e.g., fatigue or reduced feelings of vigor, is found to be a transdiagnostic symptom across many psychiatric disorders, namely major depressive disorder, social anxiety disorder, panic disorder, generalized anxiety disorder, obsessive-compulsive disorder, post-traumatic stress disorder, attention deficit hyperactivity disorder, alcohol and substance use disorder [10]. Furthermore, impaired vitality has a negative effect on both mental and physical health and is associated with cognitive deficits, decreased functioning, and a lower quality of life [11, 12, 23, 24].

Until now, there has been limited research on the associations between DM and vitality. One study investigated DM and quality of life in women with breast cancer and found that 9 of 14 DM correlated moderately positively with the fatigue subdomain (DM based on the Defenses Style Questionnaire by Hosseini; [19]). Along the same line, a review on cognitive-energetic resources associated with self-regulation – an overlapping concept with DM – summarized that these resources are finite, rapidly consumed, and slow to recover [25].

We suggest two aspects for the association of DM and vitality: Firstly, we postulate that the inhibition of awareness that is typical for immature DM is an energy-demanding process. Therefore, strong tendencies to use immature DM should lead to impaired vitality. Whereas the mainly aware experience accompanying the use of mature DM involves no inhibition of awareness, they should need no or less energy. Results from cross-sectional studies on adaptive coping mechanisms revealed no significant correlations with fatigue or vitality [26, 27], indicating that mature DM are not associated with vitality. Even though DM are supposed to reduce stress responses, an EEG study found that participants who reported greater distortions of a stressful stimulus showed more activation, indicating that immature DM involve a cognitive load, even if participants were not aware of this heightened activity during self-report [28]. This finding suggests that the use of immature DM is a resource-demanding and effortful process, going along with Freud’s theory that the maintenance of repression requires a constant effort of energy and that its resolving is a so-called economical saving (cf. psychological economy), which contributes to the psychological homeostasis and is based on the assumption that organisms only have a limited amount of energy [29]. A more recent experimental study investigating emotion regulation strategies suppression and distancing showed that these active emotion regulation strategies were perceived as effortful [30]. The second aspect that might contribute to the link between DM and vitality is that the use of immature DM might be a self-sustaining process because they do not contribute to a solution to the source of the initial anxiety or stress. Immature DM may lead to a lack of response or action in order to reduce the source of the stress or anxiety, which, therefore, might result in the persistence of the stressor, promoting the continuous need for the use of (immature) DM. Based on the assumption that the use of DM entails a higher cognitive load [28], this would also imply a greater need for energy resources, which could be accompanied by impaired vitality. Mature DM, on the other hand, lead to more awareness of one’s issues, stressors, or anxieties (see problem actuation in psychotherapy [31]), which subsequently leads to a faster resolution or elimination of the potentially energy-draining source of the conflict or stressor.

Theoretical assumptions suggest that DM and vitality are associated, but empirical evidence directly linking them is scarce. To our knowledge, no previous study has employed gold-standard questionnaires to investigate this link in a large transdiagnostic sample. To address this gap, the goal of this article is to examine the associations between DM and vitality (e.g., vigor and fatigue) in a diverse sample of adults with and without a psychiatric disorder. Based on the presented literature, we formulated the following hypotheses: H1) Higher defensive maturity is associated with more vitality. H2) Immature DM are negatively correlated with vitality. Associations of vitality with mature DM were examined exploratorily, as no strong theoretical prediction was derived.

## Methods

### Participants

This observational study was part of a larger randomized controlled clinical trial (RCT; identifier: NCT05544877). The cross-sectional data were acquired prior to randomization and the intervention phase of the RCT. The study included a planned sample of 200 participants, stratified in 100 individuals with a current or remitted psychiatric disorder and 100 individuals without a psychiatric disorder. We used the same selection criteria as in the main study. Due to the aim of capturing a broad and diverse adult population, inclusion and exclusion criteria were minimal: To participate in the study, participants had to be 18 years or older and speak German fluently. Self-reported neurological disorders that interfere with the study conduct and acute suicidality were exclusion criteria. The final sample consisted of 100 individuals without a psychiatric disorder and 100 individuals with an acute (*n* = 76) or remitted (*n* = 24) psychiatric disorder. Information about current or past psychiatric disorders was collected via self-report of participants and was not confirmed by clinical reports. Participants provided information on whether they have been diagnosed with a psychiatric disorder during their lifetime. Participants who reported a diagnosis should provide the disorder type. In addition, the administrator of each laboratory session rated the acuteness of the disorder based on current psychotherapy use and psychotropic drug use, the level of suffering, and daily life impairment with regard to functioning in social, work, and other areas of life. Sample size calculations were based on the primary outcome of a creativity intervention (reported elsewhere [32]). Participants were recruited by physicians, psychotherapists, nurses, and research staff, who informed patients of the Psychiatric Services Thurgau (PDT) about the study during group information sessions or in routine clinical interactions conducted on PDT premises. Additional recruitment strategies involved community sampling through a local newspaper, online platforms such as a student recruitment platform and social media, flyers at universities, in public places, at the Psychiatric Services Thurgau, and at outpatient psychotherapeutic offices, and reaching out through personal contacts in the city and other public locations. Participation in the study was rewarded with 30 CHF or 3 h of course credit required for psychology students.

Table 1 shows the demographic characteristics of the sample. The self-reported psychiatric diagnoses of the sample with psychiatric disorders are classified according to the DSM-5 disorders, which can be found in the Supplementary Materials (Table S3).

**Table 1:** Sociodemographic characteristics of the three subgroups and full sample

Sociodemographic variables	Current psychiatric disorder	Remitted psychiatric disorder	No disorder	Full sample
Gender				
Female	54 (71%)	20 (83%)	76 (76%)	150 (75%)
Male	21 (28%)	4 (16%)	24 (24%)	49 (24.5%)
Diverse	1 (1%)	0 (0%)	0 (0%)	1 (0.5%)
Age	36.83 (15.41)	42.08 (16.32)	38.19 (16.32)	38.12 (16.01)
Years of education	11.16 (1.95)	11.25 (2.01)	11.60 (1.70)	11.39 (1.84)
Nationality^a^				
German	37 (49%)	12 (50%)	64 (64%)	113 (57%)
Swiss	39 (51%)	14 (58%)	34 (34%)	87 (44%)

*Note. N* = 200 (current: *n* = 76, remitted: *n* = 24, no disorder: *n* = 100)

^a^ This list of nationalities is not exhaustive; a minority were dual citizens or from other countries (e.g., USA, Syria, Turkey, China)

### Study procedure

This study’s data were gathered between July 2022 and September 2023 within a randomized clinical trial investigating a creativity intervention. The study consisted of three parts: an online survey, an on-site appointment, and a five-day diary following the on-site appointment. The outcome measures used in this paper were measured in the online survey part of the study; therefore, the data are cross-sectional. The assessment hosted on SosciSurvey [33] consisted of questionnaires on DM, vitality, and demographic data, and other measures, not relevant for this sub-study. The online assessment took approximately 40 min to complete.

### Measures

#### Defense mechanisms

DM were measured using the Defense Mechanisms Rating Scale-Self-Report-30 (DMRS-RS-30) [34]. The DMRS-SR-30 is a 30-item self-report questionnaire measuring Overall Defensive Functioning (ODF), three defense categories, seven hierarchically ordered defense levels, and 28 DM on a 5-point Likert scale, with higher scores indicating a stronger use of the hierarchical level of defense mechanism. In this study, we included only items from mature and immature defense categories. Items of the neurotic defense category were omitted. Based on our theoretical assumptions, hypotheses only for mature and immature defense category, as well as their ratio, were formulated. Assessing only variables relevant to the hypotheses reduced overall participant burden, ensuring response quality. This decision was made prior to data collection, and is supported by a finding that the neurotic defense category was not significantly associated with general psychopathology, indicating that it does not depict a transdiagnostic factor [34].

Reflecting the structure of the defensive hierarchy, the DMRS-SR-30 provides several variables. ODF is an overall summary index that reveals a person’s level of defensive maturity. It is calculated as the sum of the seven (in this paper only five) defense levels weighted by the adaptiveness of the defense level in the hierarchy (action level = 1, high-adaptive = 7). The hierarchical structure of the DMRS-SR-30, showing all defense categories, levels, single mechanisms, and their corresponding items, is provided in the supplementary materials (see Table S1). To calculate the score for the mature, neurotic, and immature defense categories, all items from a category are summed up and divided by the Raw Defensive Score (RDS), the sum of all item responses, in order to receive a score for the tendency to use DM from one category relative to the multitude and strength of other DM. Since the RDS in this study consists solely of the sum of mature and immature DM items (excluding neurotic items), our mature and immature defense category scores are perfectly negatively correlated. Therefore, for the correlation tables, only the immature category score and the two immature subcategories are reported. We then conducted additional analyses with different raw scores, so without relativization to the RDS. We calculated the sum of all items of a defense category, yielding the mature raw score and the immature raw score. Moreover, we built the ratio raw score, calculated as the mean raw score of all items of the mature defense category divided by the mean raw score of all items of the Immature defense category. For the ratio raw score, we recoded the item values of the DMRS-SR-30 from 1 to 5 so as not to divide by 0. Low ratio raw scores between 0 and 1 indicate a tendency towards immature DM, while ratio raw scores above 1 indicate a tendency towards mature DM.

The English version of the DMRS-SR-30 has strong psychometric properties. Factor analyses are generally in line with expectations and only differ slightly from Vaillant’s theory of the tripartite organization of DM [35]. ODF shows excellent reliability, and acceptable-to-good reliability was reported across the three categories. Furthermore, the DMRS-SR-30 has good criterion and concurrent validity, and acceptable convergent and discriminant validity [35]. The questionnaire was translated into German by a German research group who kindly provided us with their translation. The ongoing assessment of psychometric properties of the German version and their translation procedure and quality control have not been published yet as of the date of publication [36]. The immature defense category demonstrated good internal consistency (α = 0.83), whereas the mature defense category showed moderate internal consistency (α = 0.65), which is acceptable for constructs with heterogeneous and conceptually diverse items (compare with α = 0.85 and α = 0.75 of the English version, respectively [35].

#### Vitality

The Profile of Mood States (POMS; [37] questionnaire is frequently used in clinical settings to examine psychological and somatic questions, as well as in pharmaceutical, occupational, and sports medicine research, to record the subject’s mental state [38]. The original American version consists of 65 items on a 5-point scale for the timeframe of “past week, including today”. Several shorter versions have been developed, including varying timeframes (e.g., today or right now), and also, 7-point scales have been used. From the German short version, containing 35 items on a 7-point scale across four subscales [39], we selected the three highest-loading items on the vigor and fatigue subscales, according to the validation study by Albani et al. [39]. Higher scores indicate a stronger expression of vigor or fatigue, respectively. In addition to these standard calculations, we computed a composite vitality score as the sum of vigor and inverted fatigue. The six-item version (POMS-6) used in this study showed a very good to excellent Cronbach’s alpha for the Vitality scale (α = 0.89), the Vigor subscale (α = 0.84), and the Fatigue subscale (α = 0.90; [40]).

#### Covariates

Age, gender, education, and sleep were examined as potential covariates. Sleep quality was assessed using six items from the insomnia subscale of the Inventory of Depression and Anxiety Symptoms II (IDAS-II) [41] supplemented by two additional self-developed questions assessing whether participants felt refreshed upon waking up and whether they experienced ruminating thoughts at night. These questions were administered during the on-site appointment one week after the online assessment and referred to the preceding seven days. Sleep quality was calculated as the mean of all items.

### Statistical analysis

To examine the associations of DM and vitality, bivariate correlations were calculated (Pearson’s r, two-tailed). For the regression model, linear regression was conducted. In order to adjust for the relation of sleep quality with vitality, multiple linear regression with sleep quality as an additional predictor was performed. Assumptions of linear regression (linearity, homoscedasticity, and normality of residuals) were evaluated using visual inspection of scatterplots, residual plots, and Q-Q plots. No serious violations were observed. For all DM and vitality variables, we had a complete dataset with no missing data. The covariate sleep quality had minimal missing data, with completely missing data for three participants (1.5%) and one of eight items missing for three additional participants. Participants with completely missing data were excluded from the covariate analyses. For participants with only one item missing, the sleep quality mean score was calculated with the remaining items. All analyses were conducted with SPSS 30 for Windows. We used an alpha level of 0.05 for all statistical tests. Figures were created with R and R Studio (version 2025.05.0).

## Results

The means and standard deviation of all outcome variables are listed in Table 2.

**Table 2:** Means and standard deviations of all vitality and defense mechanism scores

Variables	Current psychiatric disorder	Remitted psychiatric disorder	No disorder	Full sample
	M (SD)	M (SD)	M (SD)	M (SD)
ODF	5.14 (0.64)	5.65 (0.67)	5.54 (0.70)	5.40 (0.70)
RDS	33.02 (10.58)	25.21 (7.89)	26.24 (8.69)	28.68 (9.93)
Mature Defense Cat.	57.86 (13.81)	68.22 (14.55)	65.87 (15.42)	63.11 (15.24)
Immature Defense Cat.	42.14 (13.81)	31.78 (14.55)	34.13 (15.42)	36.89 (15.24)
Non-depressive	15.57 (6.98)	14.56 (7.68)	16.06 (9.44)	15.70 (8.35)
Depressive	26.57 (10.79)	17.22 (11.27)	18.06 (11.99)	21.19 (12.16)
Ratio raw score	1.64 (0.50)	1.91 (0.46)	1.86 (0.56)	1.78 (0.53)
POMS Vitality	21.63 (6.23)	26.71 (5.72)	27.76 (6.29)	25.31 (6.82)
POMS Fatigue	13.78 (3.87)	11.00 (3.98)	10.67 (3.87)	13.20 (3.45)
POMS Vigor	11.41 (3.42)	13.71 (2.22)	14.43 (3.13)	11.89 (4.14)

*Note. N* = 200 (current: *n* = 76, remitted: *n* = 24, no disorder: *n* = 100). ODF is the sum of the mature and immature defense level scores multiplied by a number corresponding to the position of each defense level in the hierarchy. Mature and immature defense categories are the sum of all mature or immature DM items divided by the RDS, respectively, with RDS as the sum of all mature and immature DM items. Non-depressive and depressive are subscales of the immature defense category. The ratio raw score is calculated as the mean raw score of all items of the mature defense category divided by the mean raw score of all items of the immature defense category. The POMS vitality score is the sum of the subscales vigor and inverted fatigue. Cat. = category; DM = defense mechanisms; ODF = overall defensive functioning; POMS = profile of moods scale; RDS = raw defense score

### Correlations of defense variables and vitality

**Fig. 1:**
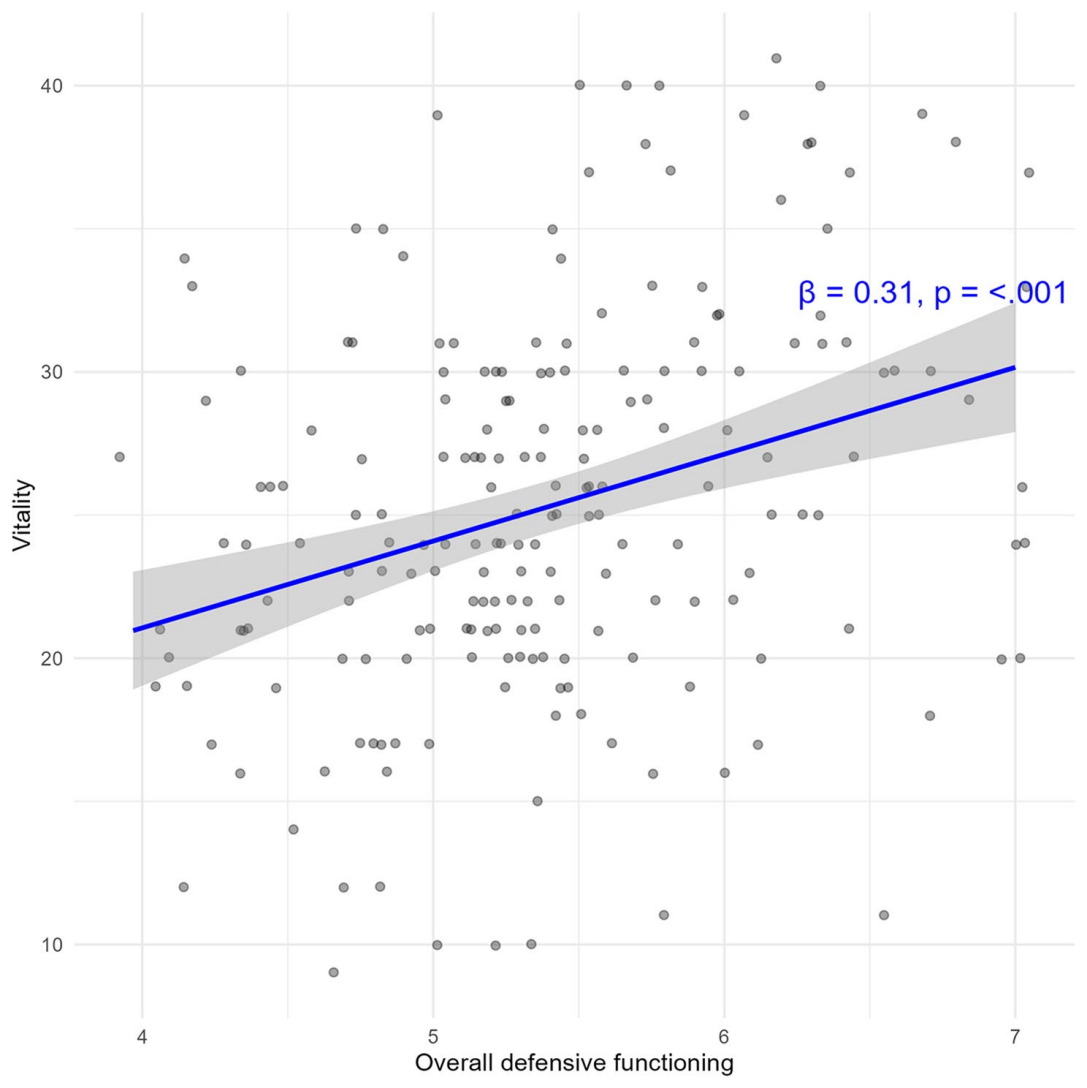
Association between overall defensive functioning and vitality. Scatterplot showing the association between overall defensive functioning (ODF) and vitality. Regression line with 95% confidence interval

**Fig. 2:**
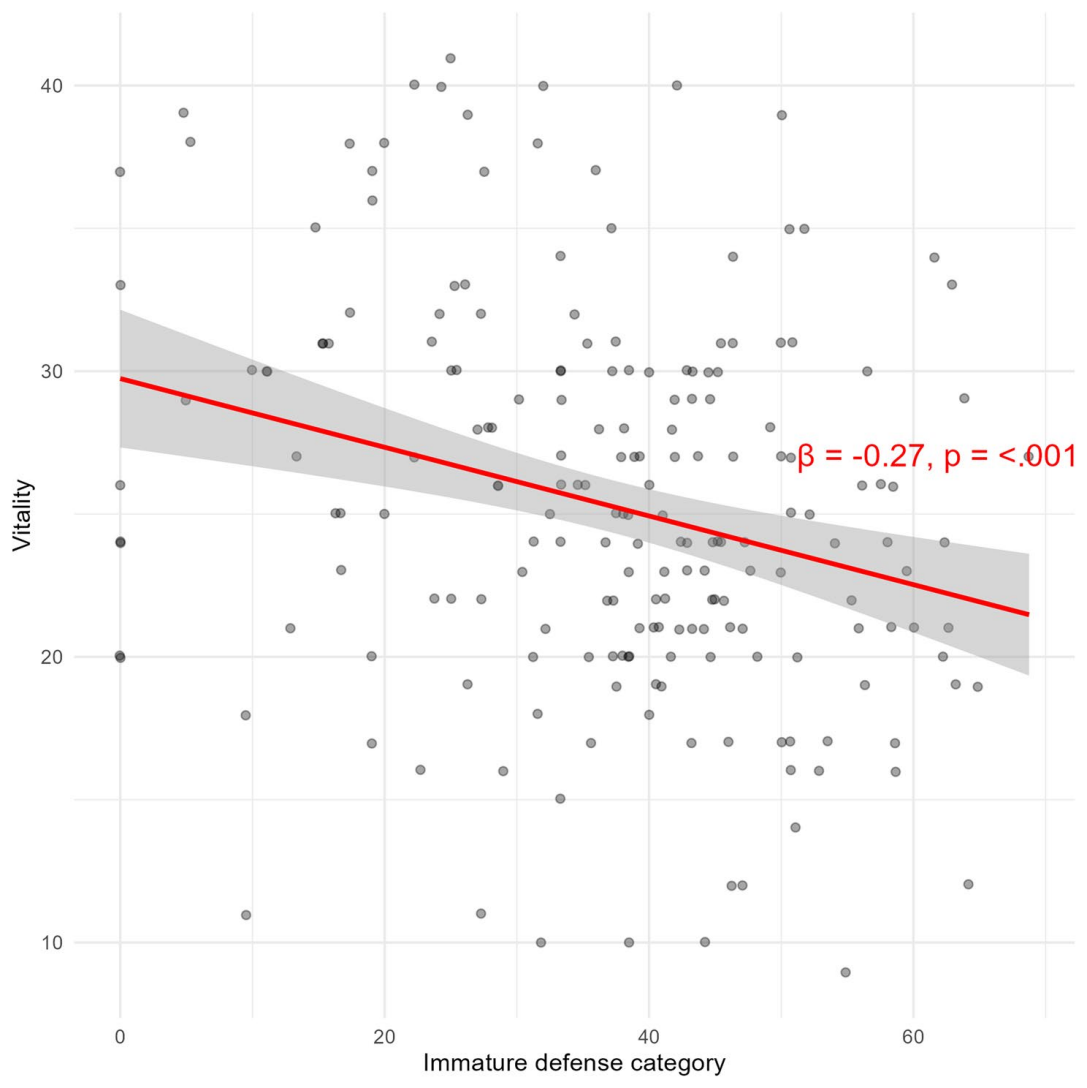
Association between immature defense category and vitality. Scatterplot showing the association between the immature defense category and vitality. Regression line with 95% confidence interval

Figure 1 shows the association between ODF and vitality. ODF, the highest level of the hierarchical defense model, was significantly positively correlated with vitality (*r* = .31, *p* < .001, 95% CI [0.18, 0.43]). The immature defense category, on the other hand, was significantly negatively associated with vitality (see Fig. 2; *r* = − .27, *p* < .001, 95% CI [0.13, 0.39]). Age, gender, education, and sleep quality were examined as potential covariates, but only sleep quality was significantly associated with vitality. The associations remained significant when adjusting for sleep quality in subsequent regression analyses, although with reduced effect sizes: ODF continued to predict vitality (*β* = 0.20, *p* = .004, B = 1.92, 95% CI [0.61, 3.22]), and sleep quality emerged as an additional significant predictor (*β* = 0.36, *p* < .001, B = 2.95, 95% CI [1.85, 4.05]). Similarly, the immature defense category remained a significant negative predictor of vitality (*β* = –0.16, *p* = .023, B = − 0.07, 95% CI [–0.13, − 0.01]), again alongside sleep quality (*β* = 0.38, *p* < .001, B = 3.10, 95% CI [2.00, 4.19]). Moreover, the multiple regression model including both the non-depressive and depressive defense subcategory as predictors, only the depressive defense subcategory significantly predicted vitality (*β* = –0.33, *p* < .001, B = − 0.18, 95% CI [–0.26, − 0.11]), whereas the non-depressive defense subcategory showed no significant association (*β* = 0.01, *p* = .873, B = 0.09, 95% CI [–0.10, 0.12]). The associations of the defense subcategories also remained robust after adjustment for sleep quality: *β* = –0.22 (*p* = .002, B = − 0.12, 95% CI [–0.20, − 0.05]) for immature depressive subcategory, and *β* = 0.03 (*p* = .614, B = 0.03, 95% CI [–0.8, 0.13]) for immature non-depressive subcategory.

### Additional exploratory analyses

Additionally, we conducted exploratory analyses, examining raw scores of the defense categories, omitting the relativization of the variables to the RDS (sum of all DM items). We examined correlations between the raw scores and vitality and conducted a linear regression analysis in which the mature and immature raw scores predicted vitality. Further exploratory scores and calculations (e.g., immature factor [35], single DM scores) with the raw defense variables, as well as additional calculations of the Energy Grid, a self-developed vitality measure, can be found in the supplementary materials (Tables S4–S8).

#### Correlations and regression model of raw defense variables and vitality

**Fig. 3:**
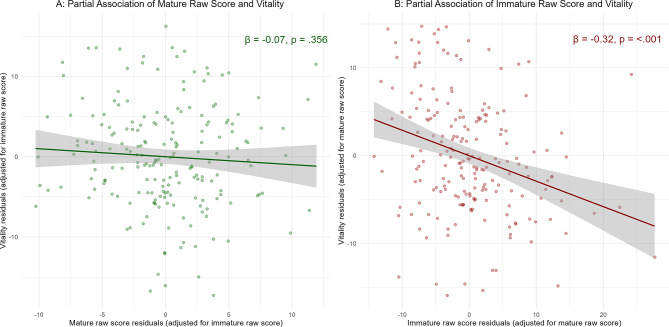
Partial associations between vitality and the mature raw score, and vitality and the immature raw score. 3A shows the association of vitality and the mature raw score, adjusted for the immature raw score. 3B shows the association of vitality and the immature raw score, adjusted for the mature raw score. The regression lines with 95% confidence intervals reflect the standardized regression coefficient of the multiple regression of mature and immature raw scores on vitality

Figure 3A and B show the partial association between each defense category’s raw scores and vitality, each plotted with its corresponding regression line. The results of the multiple regression revealed that only the immature raw score significantly predicted vitality (*β* = − 0.32, *p* < .001, B = − 0.29, 95% CI [–0.42, − 0.16]), but the mature raw score did not (*β* = − 0.07, *p* = .356, B = − 0.10, 95% CI [–0.31, 0.11]). Linear regression was not conducted with the original, relative defense variables due to multicollinearity.

The RDS, the sum of all DM items, was significantly negatively associated with vitality (*r* = − .33, *p* < .001, 95% CI [–0.45, − 0.20]). A significant negative correlation was also found for the ratio raw score (*r* = .23, *p* = .001, 95% CI [0.09, 0.35]), calculated as the mean raw score of all items of the mature defense category divided by the mean raw score of all items of the immature defense category. All associations remained robust after adjusting for sleep quality. In the regression models, immature raw score also remained a significant negative predictor of vitality (*β* = –0.19, *p* = .010, B = − 0.18, 95% CI [–0.31, − 0.04]), and the mature raw score remained not significant (*β* = –0.06, *p* = .352, B = − 0.10, 95% CI [–0.30, 0.11]). The raw defensive score also remained a significant negative predictor (*β* = –0.21, *p* = .002, B = − 0.15, 95% CI [–0.24, − 0.05]). Finally, the ratio score continued to predict vitality when adjusting for sleep quality (*β* = 0.13, *p* = .048, B = 1.70, 95% CI [0.01, 3.39]), indicating that a higher proportion of mature relative to immature defenses is associated with slightly higher vitality.

## Discussion

This study investigated the association of defense mechanisms (DM) and vitality in a transdiagnostic sample of participants with and without psychiatric disorders. Applying well-validated questionnaires to measure different hierarchical levels of DM based on the DMRS theory [34] and vitality, we found that several DM variables significantly predicted vitality, with small to moderate effect sizes. The results remained robust after adjusting for sleep quality of the past 7 days. Higher overall defensive maturity predicted more reported vitality, with a medium effect size. People with stronger relative use of immature DM reported less vitality with a medium effect size. Our findings shed light on connections between defensive functioning and vitality, both of which play a relevant role in mental and physical well-being. However, due to the cross-sectional design, no causality can be inferred.

Our first hypothesis, predicting that higher defensive maturity would be associated with greater vitality, was supported in our sample, with a medium effect size, aligning with existing results of higher defensive maturity being associated with a better mental health quality of life component (including vitality) in women with breast cancer [13]. The medium-sized negative correlation between the immature defense category and vitality supported our second hypothesis. This finding implies that people with greater use of immature DM relative to their total number and strength of all DM use report lower vitality, although the cross-sectional design does not allow for causal interpretation. Because the mature and immature defense category scores in our study are perfectly negatively correlated, their correlations with other variables are inverted. Therefore, we omitted reporting correlations for the mature defense category in this paper. Since our study design collected cross-sectional data, no conclusions about the causal direction of the two variables are possible. Therefore, future research should determine whether the maturity of a used DM determines the amount of energy demand and, in turn, a person’s vitality, whether the available energy resources determine the maturity of DM used, or whether the relationship is reciprocal. We refrained from interpreting results on the hierarchical level of the immature subcategories since the omnipotence item overly influenced the non-depressive defense subcategory in the opposite direction than expected. The DM omnipotence stands out in terms of both the direction and strength of the correlation found with vitality (see supplementary materials, Table S2). The item for omnipotence (“Did you perceive yourself as very strong, powerful, untouchable?”) has also stuck out in a recent paper testing the reliability and validity because the item did not load sufficiently high on any of the defense categories in the factor analysis [35]. We explored the reliability of the immature subscale with and without the omnipotence item. Since omitting it did not meaningfully enhance internal consistency (from α = 0.83 to α = 0.84), the item was retained in the analyses, following the scoring procedure of the questionnaire’s authors.

In our additional exploratory analyses, we looked at the raw scores of the defense categories without taking into consideration how many other DM are used alongside. First, results of the multiple regression analysis revealed that the raw immature score significantly predicted vitality while adjusting for the mature raw score and sleep quality, whereas the mature raw score did not significantly predict vitality while adjusting for the immature raw score and sleep quality. The ratio raw score predicting vitality with a small-to-medium positive effect size goes along with the other results, showing that a tendency towards immature DM is associated with less vitality, whereas a tendency towards mature DM might be associated with more vitality. This finding is also reinforced by the medium-sized negative association of the RDS with vitality. Taken together, results investigating the association of mature DM and vitality are inconclusive; therefore, our exploratory hypothesis about the association of mature defense mechanisms and vitality cannot be confirmed and needs further investigation.

As elaborated in the introduction, we offer two explanations for the association of DM and vitality. First, we assume that the inhibition of awareness of a conflict or stressor is a continuously energy-demanding process. An analogy would be a defensive wall that needs to be maintained in order to protect against threatening offenses. The paradigm of ironic effects of thought suppression might be an effect illustrating the need for continuous effort to maintain the defensive wall. This effect describes that individuals attempting to suppress an undesirable thought end up thinking of the exact thought they were trying to avoid [42]. A similar mechanism could exist for unaware content continuously trying to resurface into consciousness, resulting in a continuous need to inhibit awareness of it. The energy demand of the inhibition of awareness then leads to depletion of the available energy resources or vitality. Therefore, people using immature DM, which distort reality in a way that hinders awareness of the conflict or stressor, should report less vitality, which was supported by the results. This explanation, together with our findings, empirically supports Freud’s theory on DM as ego functions, postulating that the maintenance of repression requires a constant effort of energy, whereas resolving it grants a so-called economical saving (cf. psychological economy [29]).

Secondly, the functionality of a DM in regard to reducing the source of conflict or stress might play a role. Mature DM allow awareness of a conflict or stressor (e.g., affiliation, self-assertion) and might help find functional responses dealing with it or resolving it. The overlap of some mature DM with coping strategies supports this assumption (e.g., humor, affiliation) [15]. On the contrary, immature DM might hinder resolving or dealing with the source of conflict or stress because both emotional and cognitive parts are kept unaware, which might lead to the persistence thereof, promoting a self-sustaining process of immature DM use. Considering the opposite direction of causality, an individual might need sufficient energy resources to use mature DM due to the inherent confrontation with negative stimuli, at least temporarily (e.g., affiliation, anticipation, self-observation). Immature defenses, on the other hand, might be the automatically used option when energy sources are low since resources to deal with the cognitive or emotional demanding conflict or stressor are lacking. These two lines of argumentation about the direction of causality are not contradictory since they refer to different time frames, the first one representing a long-term process, while the second explanation covers short-term dynamics.

### Implications

Recent studies have shown that low vitality (e.g., fatigue) is a common symptom of several physical and psychiatric illnesses [10, 43]. Therefore, finding the mechanisms of action underlying transdiagnostic symptoms such as fatigue is of great societal benefit because they lay the foundation to foster the development of transdiagnostic treatments. Psychotherapeutic interventions working with the assessment and modification of DM (e.g., psychodynamic approaches) should be considered as an additional treatment for illnesses in which fatigue is an accompanying symptom, for example somatic disorders (e.g., diabetes [44]), neurocognitive disorders (e.g., multiple sclerosis [43]), and psychiatric disorders (e.g., depression, panic disorder, generalized anxiety disorder, post-traumatic stress disorder [10]). Looking at several additional defense variables, we wanted to examine whether the strength of the link between vitality and mature or immature DM differs. Our data shows that immature DM, as operationalized with the immature raw score, correlates more strongly with vitality than mature DM. Also, the regression model revealed that only the immature DM significantly predict vitality. The findings allow for tentative psychotherapeutic implications across a broad range of psychiatric disorders. However, given the small to medium effect sizes, such applications may be limited in terms of clinical significance. In order to improve vitality, interventions might prioritize reducing a person’s tendency to use immature DM. This might best be achieved by psychotherapeutic interventions or approaches working with the concept of DM, such as psychodynamic psychotherapy. Several studies have shown that the use of DM can be changed with psychotherapy [3, 45, 46]. Through learning alternative adaptive management of threatening anxieties or stressors, immature DM could be modified and might also impact fatigue and vitality. Regarding the relevance for the general population without psychiatric disorders, the understanding of how DM relate to vitality could inform interventions in the workplace to enhance stress management and performance, and in everyday life it could promote mental well-being, self-awareness, and improve interpersonal relationships. Considering the other direction of causation, interventions could focus on managing energy resources by finding ways to reduce fatigue, e.g., by improving sleep quality or nutrition. Increasing energy resources and vitality would then facilitate a person to shift from using immature DM to using mature DM. However, these causal interpretations should be regarded with care, keeping in mind the correlational nature and small to medium effect sizes of this study.

### Strengths and limitations

The study was conducted with a diverse sample of subjects with and without psychiatric disorders. Although the ratio of participants with to without psychiatric disorder is not representative of the general population, it enables a robust estimate of the association of DM and vitality due to the wide range of the sample’s reported DM use and vitality. Furthermore, the following sample characteristics limit the generalizability of the result: 75% of our participants were female and had German and/or Swiss Nationality (57% and 44%, respectively; including several dual citizens), while just a minority of participants came from other countries (e.g., USA, Syria, Turkey, China). All participants were German-speaking adults. The education level and mean age of all participants were well distributed. The most commonly reported psychiatric disorders were depressive, trauma- and stressor-related, neurodevelopmental, and personality disorders, as well as anxiety, whereas depressive disorder was predominantly prevalent. Furthermore, sampling bias may limit the generalizability of the findings. For example, participants in the study may be more motivated and have higher vitality or be more interested in psychological topics than the general population. Taken together, generalizability to other cultures and less motivated populations is limited and needs to be assessed in future studies. The study had a cross-sectional design. Hence, the results only allow interpretations of a correlational nature, not of causality. The effect sizes of the correlations examined in this study ranged from small to medium [47], which suggests that vitality is influenced by other factors as well, which should be taken into consideration in future research. Although using two gold standard questionnaires, all measures were based on self-report, increasing the risk of common-method bias. The DMRS-SR-30 is a time-efficient questionnaire measuring 28 different DM compared to other interview-based approaches. In addition, the neurotic defense category was not assessed, preventing examination of its association with vitality. Its omission also affected the computation of the mature and immature scores, which are typically scaled relative to all three categories, resulting in a perfect inverse correlation between the mature and immature defense categories. However, based on previous research showing that the neurotic defense category is not related to general psychopathology [34], we do not assume a clinically meaningful relationship with vitality as a transdiagnostic process. Future research could clarify this assumption. Furthermore, regarding the degree of unawareness of DM, the self-report questionnaire is based on the assumption that a person is at least aware of the behavior that results from the use of a DM.

### Future research

The cross-sectional results of this study provide the foundation for further research investigating causality. Future studies should employ longitudinal or interventional designs (e.g., within-subject experimental designs) to clarify directionality. For example, sleep quality could be altered in an experimental setting to manipulate vitality, or psychodynamic interventions could be used to improve DM maturity. Based on our theoretical assumptions and the current findings, we hypothesize that improvements in DM maturity will mediate increases in vitality, which in turn underlies general psychopathology, structural integration, or in other words, the level of personality functioning. Furthermore, it would be important to collect data on participants’ experienced conflicts, internal or external stressors, and perceived stress during the investigated time frame (e.g., last week) in order to adjust for actual conflicts or stressors to which a person might react to with DM. Since sleep quality is closely tied to vitality, we examined whether our findings remained robust when adjusting for sleep quality. However, future studies should further investigate how sleep quality and defense mechanisms are related, including potential causal pathways. Furthermore, future research should explore whether individuals relying heavily on immature defenses might show measurable differences in vitality-related behaviors, for example, reduced physical activity or shorter task engagement. Assessment of vitality could be complemented with biological parameters of energy metabolism such as brain mitochondrial dysregulation using broadband near-infrared spectrometry [48] or other markers of allostatic load such as cortisol. Another line of research should assess the relation between DM maturity and energy-related bodily sensations such as lightness and activation in different body parts using bodily sensation mapping tools [49].

## Conclusion

The aim of this study was to investigate the associations of defense mechanisms and vitality in a diverse sample of participants with and without psychiatric disorders. Thus far, there has been limited research on this topic. Our findings revealed that defensive maturity significantly predicted vitality (e.g., more vigor and less fatigue), even after adjusting for sleep quality. Moreover, participants reporting stronger relative use of immature DM had lower perceived vitality. Exploratory analysis using the mature and immature raw scores showed that only the immature raw score predicted vitality. This finding is in line with the theoretical assumption that the inhibition of awareness of a conflict or a stressor is an energy-demanding process and, therefore, depletes energy resources or vitality. Additionally, the findings are in line with the model that immature DM, contrary to mature DM, make it more difficult to resolve the source of conflict or stress because both its emotional and cognitive components are kept unaware, which could result in the persistence of the source of conflict or stress. Interventions in psychotherapy and at the workplace can profit from understanding the association of DM and vitality. As we found that using immature DM is a risk factor for impaired vitality and fatigue, adults with impaired vitality might profit from improvements in DM maturity. However, this hypothesis needs to be assessed in future experimental studies with a more balanced population in terms of culture and gender. In addition, psychotherapists need to be careful when working on the reality distortion of omnipotence, as it is the only immature DM with the opposite relation with vitality. Building on research identifying fatigue as a common factor of multiple psychiatric, somatic, and neurological diseases – while speculative – a wide range of disorders might profit from improvements in DM maturity.

## Supplementary Information

Below is the link to the electronic supplementary material.

Supplementary Material 1

## Data Availability

The datasets generated and analyzed during the current study are not publicly available due to confidentiality and ethical concerns regarding sensitive patient data, but are available from the corresponding author on reasonable request, provided there are no ethical considerations against it.

## Abbreviations

DM: Defense Mechanism(s)
DMRS-RS-30: Defense Mechanisms Rating Scale-Self-Report-30
ODF: Overall Defensive Functioning
POMS: Profile of Mood States
RDS: Raw Defensive Score
