# Supplementary material for: The effort of maintaining the defensive wall: is there a link between defense mechanisms and vitality?
Source: BMC Psychiatry. 2026 May 9;26:485. doi: 10.1186/s12888-026-08146-2 (PMC13292504; doi:10.1186/s12888-026-08146-2)
Supplement: Supplementary file 1 — Supplementary Material 1 [file 12888_2026_8146_MOESM1_ESM.docx]

Supplementary Materials

# Methods

## A Hierarchical Structure of the Defense Mechanisms

Table S1 shows the hierarchical structure of the DMRS-SR-30 with all defense categories, defense levels, single defense mechanisms, and their corresponding items.

**Table S1**

*Hierarchical Structure of Defense Mechanisms by Perry & Bond (2012) and the corresponding DMRS-SR-30 Items*

| Defensive category | Defense level | Defense mechanism | DMRS-SR-30 item |
| --- | --- | --- | --- |
| Mature | High adaptive | Affiliation | Did you ask for physical or emotional support while doing your best to handle the problem? |
|  |  | Altruism | Did you offer physical or psychological help to others in need? |
|  |  | Anticipation | Did you think about how you would handle difficulties that you might expect in the future? |
|  |  | Humor | Did you make humorous comments about challenging personal issues or stressful situations? |
|  |  | Self-Assertion | Did you take an active role in solving problems that arose? |
|  |  | Self-Observation | Did you reflect upon your emotional experiences and personal thoughts? |
|  |  | Sublimation | Did you try to diffuse the tension by engaging in creative activities? |
|  |  | Suppression | Did you temporarily put aside your personal needs to deal with other things that needed to be done? |
| Neurotic | Obsessional | Isolation of Affect | Did you react as if you were detached from personally relevant issues? |
|  |  | Intellectualization | Did you discuss an emotional topic in general or impersonal way, without considering or experiencing your feelings? |
|  |  | Undoing | Did you have contradictory or conflictual ideas about a topic that makes you anxious? |
|  | Neurotic | Repression | Did you have trouble remembering simple things? |
|  |  | Dissociation | Did you develop somatic symptoms, such as headache, stomach pain, or the loss of ability to do something, in response to emotional situations?  Did you feel confused, “spaced out,” or unable to talk about a distressing topic? |
|  |  | Reaction Formation | Did you have an attitude of giving much more than you received without perceiving the imbalance? |
|  |  | Displacement | Did you focus on minor or unrelated matters that distracted you away from a problem that makes you anxious? |
| Immature | Minor Image-distorting | Devaluation | Did you devalue yourself or others for your/their personal characteristics? |
|  |  | Idealization | Did you idealize yourself or others for your/their personal characteristics? |
|  |  | Omnipotence | Did you perceive yourself as very strong, powerful, untouchable? |
|  | Disavowal | Denial | Did you avoid thinking about personal problems or feelings? |
|  |  | Rationalization | Did you justify or give plausible explanations to cover up the real reasons for personal problems or stressful situations? |
|  |  | Projection | Did you have an attitude of suspiciousness or perceive others as untrustworthy, unfaithful, or manipulative? |
|  |  | Autistic (or Schizoid) Fantasy | Did you have repetitive or serial daydreams to which you retreated in lieu of real life? |
|  | Major Image-distorting | Splitting | Did you perceive others as “all good” or “all bad”?  Did you feel as if there was nothing positive or redeeming about yourself? |
|  |  | Projective Identification | Did you experience strong feelings toward someone, thinking that the other person intended to make you feel that way? |
|  | Action | Passive Aggression | Did you try to take your anger out on yourself or express it with self-harming behaviors?  Did you consciously or unconsciously try to irritate someone in indirect or annoying ways? |
|  |  | Help-Rejecting Complaining | Did you complain about how others don’t understand you or don’t really care? |
|  |  | Acting Out | Did you engage in verbal or physical fights? |

## B Energy Grid

The Energy Grid is a newly developed measurement by our lab consisting of two separate grids composed of 9 x 9 squares. The first grid maps the dimensions full of vigor to lack of vigor and inner calmness to inner restlessness. The second grid depicts the dimensions awake to tired and pleasant feelings to unpleasant feelings. Participants indicate their state on both dimensions by putting one mark in a grid. The squares are scored from 1 to 9, whereas 1 marks the extreme in the negatively connoted state (lack of vigor, inner restlessness, tired, unpleasant feelings), and 9 indicates the extreme in the positively connoted state (full of vigor, inner calmness, awake, pleasant feelings). The Energy Grid was developed based on the theoretical assumptions of the Multidimensional Mood State Questionnaire (MDBF; Steyer et al., 1997) and the methodological implementation of the Affect Grid (Russell et al., 1989). The Vitality score is built by the sum score of the value on the full of vigor to lack of vigor dimension and the awake to tired dimension. The Energy Grid is not yet validated, but Cronbach’s alpha in this study sample was acceptable for the vitality score (α = 0.75; Cronbach, 1951).

# Supplementary Results

## C Correlations of All 21 Single DM and the POMS Total Score and Scales

Correlations of all 21 single DM and the POMS total score and scales are presented in Table S2. Nine of the 21 DM showed significant associations with vitality. Five DM in the immature category had significant negative associations with vitality: Help Rejecting Complaining, Splitting Self Image, Autistic Fantasy, and Rationalization. The immature DM Omnipotence showed remarkably higher correlation coefficients compared to all other DM. Furthermore, the correlations of Omnipotence were contrary to our hypotheses for immature DM, since Omnipotence was positively associated with vitality. Four of eight mature DM correlated significantly positively with vitality: Altruism, Self-Observation, Humor, and Self-Assertion.

**Table S2**

*Correlations of Single DM and POMS Vitality Scale and Subscales*

| Defense mechanism | Defense level | POMS vitality | *p* | POMS vigor | *p* | POMS fatigue | *p* |
| --- | --- | --- | --- | --- | --- | --- | --- |
| Acting Out | 1 action | -.027 | .709 | .009 | .903 | .051 | .474 |
| **Help Rejecting Complaining** | **1 action** | **-.203** | **.004** | **-.141** | **.047** | **.217** | **.002** |
| Passive Aggression | 1 action | -.076 | .284 | -.077 | .277 | .061 | .390 |
| Splitting Object Image | 2 major image-distorting | .061 | .939 | -.004 | .952 | -.104 | .145 |
| **Splitting Self Image** | **2 major image-distorting** | **-.346** | **<.001** | **-.342** | **<.001** | **.285** | **<.001** |
| Projective Identification | 2 major image-distorting | -.101 | .155 | -.067 | .346 | .110 | .119 |
| **Autistic Fantasy** | **3 disavowal** | **-.186** | **.008** | **-.217** | **.002** | .126 | .076 |
| **Projection** | **3 disavowal** | -.135 | .056 | **-.152** | **.032** | .097 | .174 |
| **Rationalization** | **3 disavowal** | **-.166** | **.019** | **-.163** | **.021** | .137 | .054 |
| Denial | 3 disavowal | -.056 | .433 | -.059 | .406 | .043 | .549 |
| **Omnipotence** | **4 minor image-distorting** | **.479** | **<.001** | **.418** | **<.001** | **-.441** | **<.001** |
| Idealization | 4 minor image-distorting | .107 | .130 | .054 | .447 | -.132 | .062 |
| Devaluation | 4 minor image-distorting | -.090 | .206 | -.112 | .116 | .055 | .439 |
| Affiliation | 7 high-adaptive | -.120 | .089 | -.079 | .269 | .133 | .061 |
| **Altruism** | **7 high-adaptive** | **.182** | **.010** | **.209** | **.003** | -.126 | .074 |
| Anticipation | 7 high-adaptive | -.123 | .084 | -.095 | .181 | .123 | .083 |
| **Humor** | **7 high-adaptive** | **.280** | **<.001** | **.248** | **<.001** | **-.254** | **<.001** |
| **Self-Assertion** | **7 high-adaptive** | **.264** | **<.001** | **.229** | **.001** | **-.245** | **<.001** |
| **Self-Observation** | **7 high-adaptive** | **.157** | **.026** | **.158** | **.025** | -.127 | .073 |
| Sublimation | 7 high-adaptive | .118 | .096 | .118 | .095 | -.096 | .178 |
| Suppression | 7 high-adaptive | .003 | .966 | .084 | .236 | .065 | .360 |

*Note. N* = 200. Significant correlations (*p* < 0.05) in bold

Concerning the lowest hierarchical level of defenses – single DM – we found four out of eight mature DM were uncorrelated with vitality, whereas six out of 14 immature DM showed significant associations with vitality, with small to large effect sizes, which only partially confirms our hypotheses. The mature DM Affiliation, Anticipation, Sublimation, and Suppression were uncorrelated with vitality, as we hypothesized. However, participants indicating higher use of Altruism, Humor, Self-assertion, and Self-observation reported more vitality. The absent associations of the mature DM are in line with the study of McCabe et al. (2004) and Gemmell et al. (2016), which did not find correlations between vitality and adaptive coping mechanisms – a resembling concept to mature DM. The immature DM Help Rejecting Complaining, Splitting Self Image, Autistic Fantasy, Projection, and Rationalization, but not Acting Out, Passive Aggression, Splitting Object Image, Projective Identification, Denial, Idealization, and Devaluation, were negatively associated with vitality. The DM Omnipotence stands out in terms of both the direction and strength of the correlation found with vitality, showing a large effect size. The item for Omnipotence (“Did you perceive yourself as very strong, powerful, untouchable?”) has also stuck out in a recent paper testing the reliability and validity because the item did not load sufficiently high any of the defense categories in the factor analysis (Prout et al., 2022). Omnipotence is characterized by an excessively positive and inflated self-evaluation. This form of biased self-perception can function as a defensive self-enhancement strategy, temporarily reducing the subjective sense of threat and bolstering feelings of competence and control. As a result, individuals may experience a short-term increase in vitality, as their perceived abilities and emotional resources appear amplified. However, such states are typically unstable and difficult to maintain over time, possibly resulting in depressive feelings with low levels of energy at a later time point, similar to bipolar disorder.

## D Self-Reported Psychiatric Diagnoses of the Subsample With Psychiatric Disorders

**Table S3**

*Frequencies of Current and Remitted Psych. Diagnoses in Subsample With Psychiatric Disorder*

| DSM-V disorder classification | Current | Remitted |
| --- | --- | --- |
| Neurodevelopmental disorders | 14 | 0 |
| Schizophrenia spectrum and other psychotic disorders | 0 | 0 |
| Bipolar and related disorders | 2 | 0 |
| Depressive disorders | 44 | 10 |
| Anxiety disorders | 11 | 2 |
| Obsessive-compulsive and related disorders | 3 | 1 |
| Trauma- and stressor-related disorders | 16 | 6 |
| Dissociative disorders | 4 | 1 |
| Somatic symptom and related disorders | 1 | 0 |
| Feeding and eating disorders | 4 | 4 |
| Elimination disorders | 0 | 0 |
| Sleep-wake disorders | 1 | 0 |
| Sexual dysfunctions | 0 | 0 |
| Gender dysphoria | 0 | 0 |
| Disruptive, impulse-control, and conduct disorders | 0 | 0 |
| Substance-related and addictive disorders | 4 | 1 |
| Neurocognitive disorders | 2 | 0 |
| Personality disorders | 11 | 1 |
| Paraphilic disorders | 0 | 0 |

*Note.* Current: *n* = 76, remitted: *n* = 24. The self-reported diagnoses were classified according to the DSM-V disorders.

## E Correlations and Regression Model of Raw Defense Variables and Vitality (POMS)

We conducted additional analyses with different raw scores, so without relativization to the RDS. We calculated the sum of all items of a defense category, yielding the mature raw score and the immature raw score. Moreover, we built the ratio raw score, calculated as the mean raw score of all items of the Mature Defense Category divided by the mean raw score of all items of the Immature Defense Category. For the ratio raw score, we recoded the item values of the DMRS-SR-30 from 1 to 5 so as not to divide by 0. Low ratio raw scores between 0 and 1 indicate a tendency towards immature DM, while ratio raw score above 1 indicate a tendency towards mature DM.

Table S5 contains the correlations of the raw defense scores and vitality, as measured with the POMS vitality scale and subscales. The Raw Defensive Score, the sum of all DM items, was significantly negatively associated with vitality. Both the mature raw score and the immature raw score were significantly negatively associated with vitality. A significant negative correlation was also found for the ratio raw score.

**Table S4**

*Correlations of Additional Analyses With Raw DM Variables and POMS Vitality Scale and Subscales*

|  | POMS  vitality | *p* | POMS  vigor | *p* | POMS  fatigue | *p* |
| --- | --- | --- | --- | --- | --- | --- |
| RDS | -.332 | <.001 | -.305 | <.001 | .292 | <.001 |
| Mature raw score | -.174 | .014 | -.127 | .072 | .180 | .011 |
| Immature raw score | -.338 | <.001 | -.331 | <.001 | .281 | <.001 |
| Ratio raw score | .225 | .001 | .265 | <.001 | -.150 | .034 |

*Note.* *N* = 200. The RDS is the sum of all mature and immature DM items. The raw mature and immature scores are calculated as the sum of all items in the Mature and Immature Defense Category, respectively. The ratio raw score is calculated as the mean raw score of all items of the Mature Defense Category divided by the mean raw score of all items of the Immature Defense Category. The POMS Vitality score is the sum of the subscales Vigor and inversed Fatigue. DM = Defense Mechanisms; ODF = Overall Defensive Functioning; POMS = Profile of Moods Scale; RDS = Raw Defense Score.

In additional exploratory analyses, we looked at the raw scores of the defense categories without taking into consideration how many other DM are used alongside. These results indicate that both mature and immature DM are negatively associated with vitality. This means that the stronger the use of mature and immature DM is, the lower their reported vitality. However, it should be noted that the correlation with the immature DM is considerably stronger, while still a weak association. This finding is also reinforced by the negative correlation of vitality with the RDS. The positive correlation of the ratio raw score with vitality goes along with the other results, showing that a tendency towards immature DM is associated with less vitality, whereas a tendency towards mature DM is associated with more vitality.

## F Additional Defense Mechanisms Variable Calculations

In Table S6 the correlations of all single defense mechanisms raw scores with vitality are shown. Using this approach compared to the relative single defense mechanisms score revealed additional significant correlations of immature defense mechanisms and vitality. However, the significant correlations of the reported relative mature defense mechanisms were not replicated if operationalized with the defense mechanisms raw scores, but other mature defense mechanisms raw scores were significantly associated with vitality. These results indicate that the found correlations of the single mature defense mechanisms, measured with the relative score, might be overly influenced by the immature defense mechanisms integrated in the Raw Defensive Score.

**Table S5**

*Correlations of Single Defense Mechanisms Raw Item Score and POMS Vitality Scale*

| Defense mechanism | Defense level | POMS vitality | *p* |
| --- | --- | --- | --- |
| **Acting out** | **1 action** | **-.152** | **.032** |
| **Help Rejecting Complaining** ^a^ | **1 action** | **-.301** | **<.001** |
| **Passive aggression 1** | **1 action** | **-.246** | **<.001** |
| Passive aggression 2 | 1 action | -.074 | .299 |
| Splitting Object Image | 2 major image-distorting | -.091 | .198 |
| **Splitting Self Image** ^a^ | **2 major image-distorting** | **-.406** | **<.001** |
| **Projective Identification** | **2 major image-distorting** | **-.195** | **.006** |
| **Autistic Fantasy** ^a^ | **3 disavowal** | **-.286** | **<.001** |
| **Projection** ^a^ | **3 disavowal** | **-.246** | **<.001** |
| **Rationalization** ^a^ | **3 disavowal** | **-.285** | **<.001** |
| **Denial** | **3 disavowal** | **-.183** | **.009** |
| **Omnipotence** ^a^ | **4 minor image-distorting** | **.348** | **<.001** |
| Idealization | 4 minor image-distorting | -.053 | .455 |
| **Devaluation** | **4 minor image-distorting** | **-.205** | **.004** |
| **Affiliation** | **7 high-adaptive** | **-.247** | **<.001** |
| Altruism ^a^ | 7 high-adaptive | -.064 | .368 |
| **Anticipation** | **7 high-adaptive** | **-.308** | **<.001** |
| Humor ^a^ | 7 high-adaptive | .099 | .162 |
| Self-Assertion ^a^ | 7 high-adaptive | .071 | .317 |
| Self-Observation ^a^ | 7 high-adaptive | -.058 | .418 |
| Sublimation | 7 high-adaptive | -.028 | .698 |
| **Suppression** | **7 high-adaptive** | **-.203** | **.004** |

*Note. N* = 200. Significant correlations are in bold.
^a^ defense mechanisms that correlated significantly with vitality, when measured as relative score (divided by the raw defense score, which is the sum of all item answers).

Table S7 additionally shows the correlation of vitality and the immature defenses calculated based on factor analysis (omitting three defense mechanisms from the immature category; Prout et al., 2022). The immature defense factor shows significant negative correlations to vitality. Moreover, correlations of vitality and the defense levels are shown, which are a hierarchical level below the defense categories. All defense levels showed significant correlations except level 4 minor image-distorting. This is likely due to the Omnipotence item in this level, which shows a remarkably different correlation coefficient in both strength and direction (as discussed in the article).

**Table S6**

*Correlations of Further Original Defense Mechanisms Variables and POMS Vitality Scale*

| Defense level | POMS vitality | *p* |
| --- | --- | --- |
| Immature factor | -.311 | <.001 |
| 1 action | -.234 | .001 |
| 2 major image-distorting | -.290 | <.001 |
| 3 disavowal | -.279 | <.001 |
| 4 minor image-distorting | .216 | .002 |
| 7 high-adaptive | .269 | <.001 |

*Note. N* = 200. Defense levels relative to raw defensive score. Non-depressive and depressive are subscales of the immature DM category and for the variable factor immature the defense mechanisms autistic fantasy, denial & omnipotence are not included, based on psychometric evaluation by Prout (2022).

## G Correlations of Defense Variables and Vitality, as measured with the Energy Grid

As exploratory analyses, we also looked at the associations of the defense variables with vitality, measured with the Energy Grid. Results are shown in Table S8. We found significant positive associations of ODF with vitality. Immature Defense Category, and the Non-depressive Defense Subcategory were not significantly associated with vitality, whereas the Depressive Defense Subcategory was significantly negatively correlated with vitality.

**Table S7**

*Exploratory Correlations of the Defense Variables and Vitality, as Measured With the Energy Grid*

|  | EG vitality | *p* |
| --- | --- | --- |
| ODF | **.157** | **.027** |
| RDS | **-.155** | **.029** |
| Immature category | -.127 | .074 |
| Non-depressive | .021 | .768 |
| Depressive | **-.173** | **.014** |

*Note.* *N* = 200. ODF is the sum of the mature and immature defense level scores multiplied by a number corresponding to the position of each defense level in the hierarchy. The immature category variable is the sum of all mature or immature DM items divided by the RDS, respectively, with RDS as the sum of all mature and immature DM items. Since the mature and immature defense category scores are perfectly negatively correlated in our study, we report only the immature defense category. Non-depressive and depressive are subscales of the immature defense category. The EG vitality score is the sum of the subscales vigor and inversed fatigue. DM = defense mechanisms; EG = Energy Grid; ODF = overall defensive functioning; RDS = raw defense score.

In these explorative analyses of the association of DM with vitality using the Energy Grid, ODF, RDS, and the depressive defense subcategory correlated significantly, but very weakly, with this vitality measure. These results, therefore, only partially confirm our hypothesis, which may be due to the reduced reliability of this short questionnaire. Furthermore, we found that people reporting using a greater variety of DM and a higher frequency of use, as indicated by a higher score on RDS, show lower vitality. This could mean that if a person uses DM often and/or uses a broad variety of DM they feel less vitality.

## H Additional Calculations With Vitality, as Measured With the Energy Grid

The correlations of the raw defense scores and vitality, as measured with the Energy Grid are reported in Table S9. The RDS, the sum of all DM items, and vitality were significantly negatively associated. The immature raw score correlates significantly negatively with vitality, whereas no significant correlations were found for vitality and the mature raw score as well as the ratio raw score.

**Table S8**

*Correlations of Additional Analyses With Raw DM Variables and Vitality, as Measured With the Energy Grid*

|  | EG vitality | p |
| --- | --- | --- |
| RDS | -.155 | .029 |
| Mature raw score | -.086 | .225 |
| Immature raw score | -.154 | .029 |
| Ratio raw score | -.096 | .176 |

*Note.* *N* = 200. The RDS is the sum of all mature and immature DM items. The raw mature and immature scores are calculated as the sum of all items in the mature and immature defense category, respectively. The ratio raw score is calculated as the mean raw score of all items of the immature defense category divided by the mean raw score of all items of the mature defense category. The EG Vitality score is the sum of the scales awake-tired and full of energy-lack of energy. DM = defense mechanisms; EG = Energy Grid; RDS = raw defense score.

Taking into consideration that the mature raw score does not correlate with the EG vitality measure, but the immature raw score does, although very weakly, we can assume that the correlation of RDS with vitality is mainly due to the immature DM. This goes along with the other results suggesting that immature DM have a greater association with vitality then mature DM.

## References

Cronbach, L. J. (1951). Coefficient alpha and the internal structure of tests. *Psychometrika*, *16*(3), 297–334. https://doi.org/10.1007/BF02310555

Gemmell, L. A., Terhorst, L., Jhamb, M., Unruh, M., Myaskovsky, L., Kester, L., & Steel, J. L. (2016). Gender and Racial Differences in Stress, Coping, and Health-Related Quality of Life in Chronic Kidney Disease. *Journal of Pain and Symptom Management*, *52*(6), 806–812. https://doi.org/10.1016/j.jpainsymman.2016.05.029

McCabe, M. P., McKern, S., & McDonald, E. (2004). Coping and psychological adjustment among people with multiple sclerosis. *Journal of Psychosomatic Research*, *56*(3), 355–361. https://doi.org/10.1016/S0022-3999(03)00132-6

Perry, J. C., & Bond, M. (2012). Change in Defense Mechanisms During Long-Term Dynamic Psychotherapy and Five-Year Outcome. *American Journal of Psychiatry*, *169*(9), 916–925. https://doi.org/10.1176/appi.ajp.2012.11091403

Prout, T. A., Di Giuseppe, M., Zilcha-Mano, S., Perry, J. C., & Conversano, C. (2022). Psychometric Properties of the Defense Mechanisms Rating Scales-Self-Report-30 (DMRS-SR-30): Internal Consistency, Validity and Factor Structure. *Journal of Personality Assessment*, *104*(6), 833–843. https://doi.org/10.1080/00223891.2021.2019053

Russell, J. A., Weiss, A., & Mendelsohn, G. A. (1989). Affect grid: A single-item scale of pleasure and arousal. *Journal of Personality and Social Psychology*, *57*(3), 493.

Steyer, R., Schwenkmezger, P., Notz, P., & Eid, M. (1997). The multidimensional mental state questionnaire (Mehrdimensionale Befindlichkeitsfragebogen (MDBF)): Manual. *Göttingen, Germany: Hogrefe*.
